# Intractable hyperkalemia caused by hepatic infarction developed during laparoscopic gastrectomy in a patient with end-stage renal failure: a case report

**DOI:** 10.1186/s40981-019-0280-8

**Published:** 2019-09-10

**Authors:** Eriko Takeyama, Nobuyuki Nishimura, Eizo Amano, Hiromi Shibuya

**Affiliations:** 1 Department of Anesthesiology, National Hospital Organization Osaka National Hospital, 2-1-14, Hoenzaka, Chuo-Ku, Osaka City, Osaka, 540-0006 Japan; 2 Department of Anesthesiology, Osaka Police Hospital, 10-31, Kitayama-cho, Tennoji-Ku, Osaka City, Osaka, 543-0035 Japan

**Keywords:** Laparoscopic gastrectomy, Liver infarction, Hyperkalemia, Hemodialysis

## Abstract

**Background:**

Patients with renal failure are susceptible to electrolyte disturbances including life-threatening hyperkalemia, and intraoperative hepatic damage exacerbates it. We report a case on hemodialysis who developed intraoperative remarkable hyperkalemia caused by hepatic damage during laparoscopic gastrectomy.

**Case presentation:**

A 48-year-old man underwent laparoscopic gastrectomy for gastric cancer. He had been on hemodialysis for chronic renal failure. Serum K^+^ continued to increase to a maximum level of 7.4 mEq/L, despite the infusion of glucose with insulin during surgery. Postoperative computed tomography revealed hepatic infarction. Combined with increased hepatic enzymes, hepatic infarction caused by intraoperative mechanical traction would have exacerbated hyperkalemia.

**Conclusions:**

We report a case on hemodialysis who developed intraoperative hyperkalemia due to hepatic damage. Our case highlights hepatic damage during laparoscopic gastrectomy as a potential cause of hyperkalemia.

## Background

Patients with renal failure are susceptible to developing metabolic acidosis and hyperkalemia perioperatively. Liver surgery or liver ischemia often causes hyperkalemia, due to potassium (K^+^) release from damaged hepatocytes [1]. Liver damage can happen during laparoscopic gastrectomy (LG) from intraoperative manipulation such as liver retraction to maintain a sufficient surgical field [2]. Clinically, retraction-related liver injuries are usually mild and reversible. Retraction-related severe hepatic infarction as a complication following LG is not widely known [3, 4]. This is the first report of severe intraoperative hyperkalemia due to retraction-related hepatic infarction during LG. Our case also highlights the fact that renal failure potentiates hyperkalemia caused by liver damage during LG.

## Case presentation

A 48-year-old man (height 168 cm, weight 80 kg) with gastric cancer was scheduled for laparoscopic gastrectomy. He was on maintenance dialysis for chronic renal failure. His medical history included coronary artery bypass grafting for angina. Blood tests were done a day before surgery and prior to maintenance hemodialysis showed mild hyperkalemia (serum K^+^ 5.4 mEq/L). Serum K^+^ was not rechecked after hemodialysis.

In the operating room, after placing a thoracic epidural catheter, we induced general anesthesia by intravenous administration of propofol 60 mg, fentanyl 100 μg, and rocuronium 70 mg. A central venous catheter was placed via the internal jugular vein. Anesthesia was maintained with oxygen (40 to 50%), air and desflurane (4 to 5%); and a continuous infusion of remifentanil (0.1–0.2 μg/kg/min). Bolus 1.5% lidocaine epidural infusion and intravenous fentanyl infusion were added as needed. Circulation was supported by continuous nicorandil infusion and bolus phenylephrine. In addition to standard monitors, continuous arterial pressure was monitored via the radial artery.

Surgery was performed in the reverse Trendelenburg position. An hour into surgery, his arterial blood gas (ABG) analysis showed hyperkalemia serum K^+^ =6.3 mEq/L (Table 1). The patient had received K^+^ free infusion since arriving in the operating room until then. The cause for hyperkalemia was unclear at that point in time. In the surgical field, the liver was enlarged and had been retracted for optimal exposure.

**Table 1:** Arterial blood gas data

		Surgery time (h)						Postoperative time (h)							
		1	3.5	5.5	6.5	9.5	11	0	3	7	12	14	20	24	48
pH		7.441	7.357	7.365	7.329	7.337	7.335	7.293	7.363	7.401	7.429	7.443	7.376	7.417	7.379
pCO2	mmHg	36.2	40.6	39	39	36.1	36.4	41.5	40.6	37.1	35.7	35.2	45.7	36.6	43.7
pO2	mmHg	146.8	202.9	200.2	173.1	158.5	149.4	98.6	78.3	85.4	75.6	104	34.1	93	104
HCO3	mEq/L	24.1	22.3	21.8	20.1	18.9	19	19.5	22.5	22.5	23.2	23.7	26.1	23.2	25.2
BE	mEq/L	0.2	− 3	− 3.2	− 5.4	− 6.2	− 6.2	− 6.2	− 2.2	− 1.4	− 0.3	0.3	1.2	− 0.6	0.5
tHb	g/dL	11.3	11.8	11.4	10.3	11.3	10.9	10.9	10.9	10.2	10.8	10.6	10.0	9.5	8.8
Na^+^	mEq/L	133.9	132.2	132.0	133.5	130.7	132.4	131	131	132	132	134	131	131	132
K^+^	mEq/L	6.3	6.8	7.0	6.5	7.4	7.2	6.7	8	6.9	6.6	3.8	7.1	5.8	4.6
Lac	mg/dL	7.1	8.5	10.4	9.4	10.1	14.7	27	18	23	14	14	32	18	8

Glucose-insulin infusion started from the fourth hour of surgery. *BE* base excess, *tHb* total hemoglobin, *Hct* hematocrit, *Glu* blood glucose, *Lac* serum lactate

ABG analysis during the third hour of surgery showed a further increase in serum K^+^ to 7.0 mEq/L (Table 1). Hence, we initiated an infusion of 50% glucose at 5 mL/h with insulin at 0.2 U/h. As serum K^+^ remained high, we increased the ratio and volume of the infusion gradually to 50% glucose at 20 mL/h with 2 U/h of insulin. When the K^+^ level exceeded 7 mEq/L during the fifth hour of surgery, sodium bicarbonate (120 mg) was administered. We reported the hyperkalemia to surgeons and discussed to arrange an emergency postoperative hemodialysis. They did not report any abnormal changes that could be the cause of the hyperkalemia in the surgical field, including color changes to the liver. The K^+^ level continued to remain high despite remedial measures. From the eighth hour onwards, the lactate level also increased (Table 1). The total surgical duration was 601 min, and anesthesia time was 743 min.

The patient received a total of 900 mL of normal saline and 500 mL of 5% albumin during the procedure. His estimated blood loss was 350 mL. The patient was hemodynamically stable throughout the operation, with no abnormal findings on electrocardiogram (ECG). After the conclusion of the surgery, the patient was extubated in the operating room and transported to the intensive care unit.

**Fig. 1:**
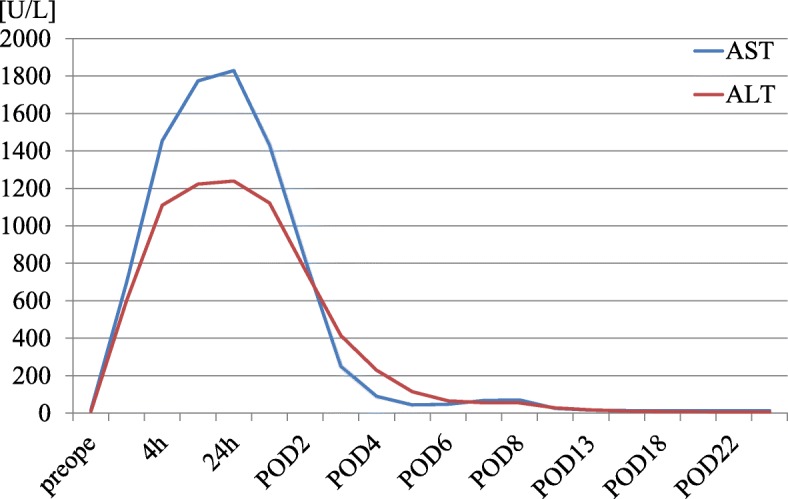
Pre- and post-operative changes in serum liver enzymes. AST, aspartate aminotransferase; ALT, alkaline aminotransferase

**Fig. 2:**
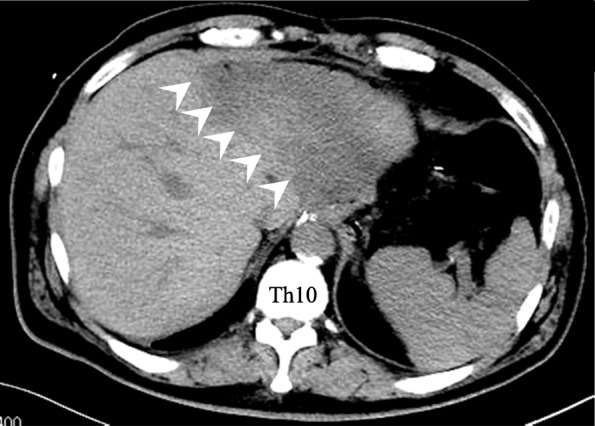
Computed tomography on the fifth postoperative day. CT revealed a low-density area in the lateral segment of the liver indicated by arrowheads, suggesting infarction

Immediate postoperative blood analysis showed a significant rise in aspartate aminotransferase (AST) and alanine aminotransferase (ALT) (Fig. 1), suggestive of liver damage, most probably due to intraoperative liver retraction. We initiated glycyrrhizin infusion for suspected liver damage. Serum K^+^ decreased marginally to 6.7 mEq/L (Table 1) because of continuous glucose-insulin infusion, and thus an emergency postoperative hemodialysis was avoided. However, we resumed hemodialysis on the first postoperative day (POD 1). Serum K^+^ was 7.0 mEq/L (Table 1) despite hemodialysis and so he received continuous hemodiafiltration from POD 1 to POD 4. Serum K^+^, AST, and ALT peaked on POD 1 and then improved gradually with the above treatment measures (Fig. 1). However, the patient developed a high-grade fever (39 °C) on POD 5. Emergent computed tomography (CT) showed a low-density area in the left lobe of the liver (Fig. 2), suggestive of hepatic infarction. Treatment with antibiotic agent was initiated for presumed infection of the infarcted liver. The patient responded well to treatment. He was discharged 27 days after surgery.

## Discussion

Intraoperative hyperkalemia can be caused by excessive potassium administration by infusion or transfusion, or by decreased potassium excretion as in renal failure, or by the movement of intracellular potassium out of the cells due to acidosis or tissue injury.

In our case, we experienced an unexpected increase in serum K^+^ an hour after the initiation of the surgery. We attributed his renal failure as the possible cause for the rise in serum K^+^. There is also the possibility that the hemodialysis might have been insufficient, or the patient might have ingested foods or drinks containing K^+^ before surgery. All of these could have increased serum K^+^ before the initiation of surgery. During surgery, the patient received K^+^ free infusion, did not receive any transfusion or K^+^ releasing drug such as succinylcholine. A mild decrease in pH was observed early in the surgery (Table 1), but it is unlikely that it directly caused the hyperkalemia. Even with careful monitoring and treatment, serum K^+^ level continued to increase. We were not able to determine the cause of hyperkalemia intraoperatively. The deranged liver panel obtained immediately after surgery pointed to liver damage as a potential cause of hyperkalemia.

Liver damage is a known complication of liver surgery and is also reported to be caused by LG [2]. Although LG has several advantages over conventional surgery, postoperative liver dysfunction remains an unwanted complication. However, severe liver damage such as hepatic infarction by LG is rare. Hence, we did not consider the possibility of liver damage in our case. Recent studies have implicated mechanical liver retraction to be a major cause of hepatic injury [2, 4, 5]. Liver retraction is essential for optimal anatomical exposure during LG. Such retraction increases pressure on the liver parenchyma, by compressing it between the retractor blade and the diaphragm. This increase in pressure is potentiated by positioning the patient in reverse Trendelenburg for long durations.

Though the physiological mechanism underlying the development of retraction-related liver damage is not fully clear, its causes can be classified broadly into two types based on the review of available literature. The first type is the result of a retraction-related parenchymal fracture or tear, and is caused by direct physical injury from the retractor blade [3]. Most such parenchymal injuries are identified at the time of their occurrence, but could also present postoperatively due to a slowly developing subcapsular hematoma [4]. In our case, the postoperative CT showed no such hematoma. The second type occurs from impaired blood flow to the parenchyma because of prolonged compression. Such pressure-related injuries are usually temporary. However, depending on the duration of retraction and the amount of liver tissue trapped, the impaired blood flow can be severe enough to cause parenchymal infarction. Kitagawa et al. [5] reported a case of retractor-related hepatic infarction following gastric surgery, in which postoperative angiography showed a reduction in blood flow to the lateral side of the liver despite the preservation of the hepatic artery. This shows that a decrease in portal blood flow due to prolonged retractor use can also cause an infarction. In our case, the impaired blood flow due to compression by retractor, along with risk factors such as an enlarged liver and a prolonged reverse Trendelenburg position, caused pressure-related liver damage and infarction.

The damaged and dying liver cells release their intra-cellular K^+^ into the blood. This happens in acute hepatic necrosis and in those with hepatic damage due to compression by surgical manipulation [6]. In animal studies, blocking the hepatic blood flow releases K^+^ from ischemic hepatic cells and causes hyperkalemia [7]. Patients who are unable to handle the additional potassium load due to renal insufficiency might experience severe and intractable hyperkalemia.

Hyperkalemia has definite effects on cardiac conduction. Severe hyperkalemia requires urgent treatment with pharmacological agents or early dialysis. In our case, we arranged emergency postoperative hemodialysis since serum K^+^ exceeded 7 mEq/L during surgery. The high-dose glucose-insulin infusion eventually decreased serum K^+^ and no abnormal ECG changes were observed. Postoperative emergency hemodialysis may cause circulatory changes resulting in cerebrovascular and myocardial ischemia. When serum K^+^ reduced in our case, it was reasonable to avoid emergent hemodialysis to minimize circulatory fluctuations in the acute phase after surgery.

During LG, surgeons should be aware of liver discoloration in order to prevent serious liver injury before it occurs. Kitajima et al. [8] have suggested that changing the retractor position or releasing it intermittently could prevent liver damage during LG. The anesthesiologist and operating room nurses can communicate periodically with the surgeon to release or move the retractor intermittently when the operation time increases. Careful monitoring of all hematologic and electrolyte abnormalities is also recommended during LG. In acute hepatic necrosis, laboratory blood test might detect hyperkalemia prior to or concomitant with marked elevations in hepatic enzymes. All these might be useful in preventing further elevation of K^+^ during surgery.

Our case report emphasizes the need for awareness of liver damage and hyperkalemia during LG, especially in patients with renal failure. During LG, when unexplained hyperkalemia is observed, it might be beneficial to look for causes in the surgical field to prevent further elevation of serum K^+^.

## Data Availability

The datasets analyzed in this article are included in the article.

## Abbreviations

ABG: Arterial blood gas
ALT: Alanine aminotransferase
AST: Aspartate aminotransferase
CT: Computed tomography
ECG: Electrocardiogram
K^+^: Potassium
LG: Laparoscopic gastrectomy
POD: Postoperative day
